# IVUS Validation of Patient Coronary Artery Lumen Area Obtained from CT Images

**DOI:** 10.1371/journal.pone.0086949

**Published:** 2014-01-29

**Authors:** Tong Luo, Thomas Wischgoll, Bon Kwon Koo, Yunlong Huo, Ghassan S. Kassab

**Affiliations:** 1 Department of Biomedical Engineering, Indiana University Purdue University Indianapolis, Indianapolis, Indiana, United States of America; 2 Department of Computer Science and Engineering, Wright State University, Fairborn, Ohio, United States of America; 3 Department of Internal Medicine, Seoul National University College of Medicine, Seoul, South Korea; 4 Department of Surgery, Indiana University Purdue University Indianapolis, Indianapolis, Indiana, United States of America; 5 Department of Cellular and Integrative Physiology, Indiana University Purdue University Indianapolis, Indianapolis, Indiana, United States of America; University of Arizona, United States of America

## Abstract

**Aims:**

Accurate computed tomography (CT)-based reconstruction of coronary morphometry (diameters, length, bifurcation angles) is important for construction of patient-specific models to aid diagnosis and therapy. The objective of this study is to validate the accuracy of patient coronary artery lumen area obtained from CT images based on intravascular ultrasound (IVUS).

**Methods and Results:**

Morphometric data of 5 patient CT scans with 11 arteries from IVUS were reconstructed including the lumen cross sectional area (CSA), diameter and length. The volumetric data from CT images were analyzed at sub-pixel accuracy to obtain accurate vessel center lines and CSA. A new center line extraction approach was used where an initial estimated skeleton in discrete value was obtained using a traditional thinning algorithm. The CSA was determined directly without any circular shape assumptions to provide accurate reconstruction of stenosis. The root-mean-square error (RMSE) for CSA and diameter were 16.2% and 9.5% respectively.

**Conclusions:**

The image segmentation and CSA extraction algorithm for reconstruction of coronary arteries proved to be accurate for determination of vessel lumen area. This approach provides fundamental morphometric data for patient-specific models to diagnose and treat coronary artery disease.

## Introduction

With the enormous advances in computational science and medical imaging technologies in the past decade, patient-specific models are becoming more common to aid in diagnosis and therapeutics. Computational modeling of coronary artery disease requires accurate measurement of cross-sectional area (CSA) and length of the 3D vessels. Accordingly, the combination of imaging (e.g., computed tomography, CT) and computational simulations have been used to investigate the role of biomechanical factors in vascular disease [1]–[5] and vascular surgeries [6]–[8]. Computational models have also been used for device simulations [9]–[11]. These developments are at an early stage and idealized arterial models are typically used (e.g., straight tubes). The simulations have largely not been coupled with patient-specific, image-based vascular models. This is an important limitation that requires an accurate and reproducible algorithm to faithfully reconstruct the coronary anatomy from medical images.

Since image segmentation can be a tedious task to reconstruct the 3D geometric structure, much effort in CT image analysis has been devoted to develop a fully automatic or semi-automatic segmentation approaches. Thus, labor saving methodology that retains accuracy is a major research topic [12]–[14]. In addition to development of automatic methods for cardiovascular image segmentation, the validation of the segmentation accuracy is important for image analysis and computational modeling to ensure the faithful reconstruction of the anatomical structure which in turn dictates the accuracy of hemodynamic predictions.

*In vitro* validation of CT image segmentation using microscopy has been performed by our group and the agreement error was found to be <10% for lumen diameter [15]. Voros S et al. recently reported a validation study of coronary CT anatomy with IVUS [16] with errors of 21% in lumen area reconstructed from CT images and overestimated diameter stenosis by 39%. Clearly, these errors are unacceptably large and would propagate significant errors in hemodynamic and mechanical parameters in model simulations.

Here, we used IVUS to validate CT image segmentation for extraction of coronary morphometry with focus on accuracy of lumen area for normal and stenotic vessels. A new center line extraction method was proposed to improve the geometric accuracy at sub-pixel level. Based on CT images, the center line was extracted and found to accurately reproduce the vessel axis. Subsequent to centerline extraction, the lumen CSA of coronary arteries in normal and stenotic vessels was validated by IVUS.

## Methods

### Image Data of Coronary Arteries

The clinical study protocol was approved by the ethics committee of Seoul National University Hospital and all participants gave written consent to participate in this study. Five patients with coronary lesions in 11 major coronary arteries (left anterior descending artery, LAD; right coronary artery RCA; and left circumflex artery, LCX) were scanned with CT. The image segmentation results from CT imagery were validated by IVUS (“gold standard”). The patients underwent 64-slice CCTA during a routine health check. IVUS and angiography were performed in a standard fashion. IVUS analysis were performed by an independent core laboratory at Seoul National University Cardiovascular Center [17]. The CT and IVUS data were saved as DICOM while angiography data were saved using the JPEG format. The CT images provided the images for 3D structure reconstruction and the IVUS data provided the 2D cross sections for each point along the center line. Angiography was used to identify landmarks to overlap the CT and IVUS data.

**Figure 1:**
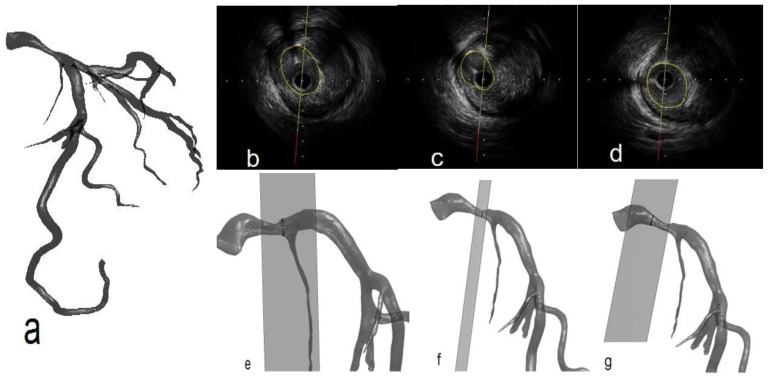
a) Example of CT image segmentation and the 3D geometric reconstruction of one LAD. **b**) IVUS example from one frame of the LAD artery. Lumen region is marked in polygon. **c**) A frame of IVUS. **d**) A Third frame. **e**) The corresponding CSA location to IVUS frame in Figure 1b. **f**) A second CSA. **g**) A Third CSA.

The entire 3D geometric reconstruction from CT image segmentation is shown in **Figure 1a**. The results of IVUS from one LAD were represented in the form of individual images as seen in **Figures 1b, 1c, 1d**. After the lumen region was outlined using a polygon curve, the geometric information of the CSA was extracted and the length was recorded from the same point of interest. The CSA was used to validate the reconstruction of CT image analysis. One or two angiographic images were used for each artery to ensure identification of the same points of interest in CT and IVUS. In this investigation, angiography in specific viewpoints was only used to provide 3D spatial structure reference for IVUS. The corresponding 3D CSA locations are demonstrated in **Figures 1e, 1f, 1g**.

### CT Image Analysis

Image analysis was composed of several image processing steps: image segmentation, 3D reconstruction, center line extraction and CSA computation. The CT image segmentation was based on local features. The initial automatic processing was complemented by manual modification. By localization at several slices, some seed points were inputted manually or by computer processing. As high intensity levels inside the vessels are stable near the skeleton region, initial seeds were located with a higher threshold, so that the skeleton region can be extracted. Region growing was then used to refine the object region based on the local histogram which was computed from pixels within a sphere. This spherical neighborhood region was centered on initial seeds. By dividing the histogram bins into target and non-target objects, feature-centers were formed and used in a further feature-clustering algorithm. In some cases, the image quality was not satisfactory with automatic processing, such that manual intervention was used to remove some adhered regions in the vessels.

**Figure 2:**
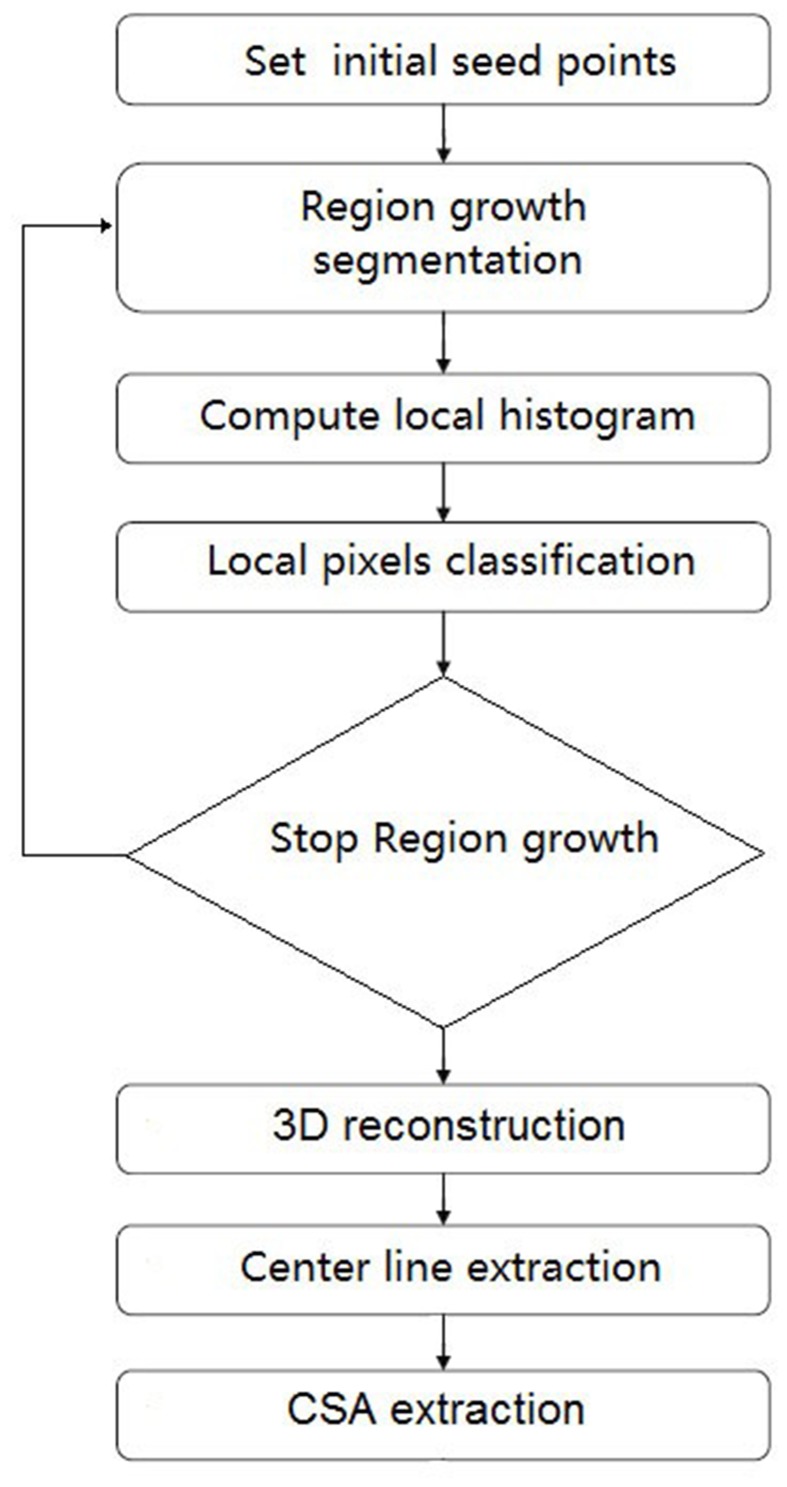
A schematic flow chart for the solution algorithm.

Data from CT images contain intensity values which are measured in Hounsfield Units (HU), which are a linear transformation of the attenuation coefficient measurement in which the radiodensity of distilled water at standard pressure and temperature maps to a HU of zero whereas the radiodensity of air at standard pressure and temperature amounts to −1000 HU. The vessels were first segmented from other background tissue. Calcific stenosis has intensity values typically >700 HU, which is relatively high as compared to the radiodensity of a normal vessel. A deconvolution method was used as a preprocessing procedure to overcome the point diffusion effect between the normal vessel and stenosis. Further classification steps, such as simple threshold and local maximal gradient, were applied for these regions to identify a stenosis. The 3D surface mesh of the vessel was reconstructed from segmentation result by classical Marching Cubes technique [18]. After the mesh surface was reconstructed, curvature smoothing was used to further smooth the lumen surface mesh. The center line was first computed by image thinning and refined by Bézier interpolation to obtain the center line accurately at a sub-voxel level. A flow chart of image processing steps is outlined in **Figure 2**. A more detailed description can be found in Appendix S1.

## Results

In the five patients imaged, 11 vessels and over 1,300 IVUS cross-sectional images were sampled, and over 400 originally thinned CT points were interpolated to yield matching points between IVUS and CT. Although the data were extracted from only five patients, multiple positions were sampled from each patient to provide 1,314 data for comparison between CT and IVUS. Hence, the sample size was sufficiently powered for statistical analysis.

**Figure 3:**
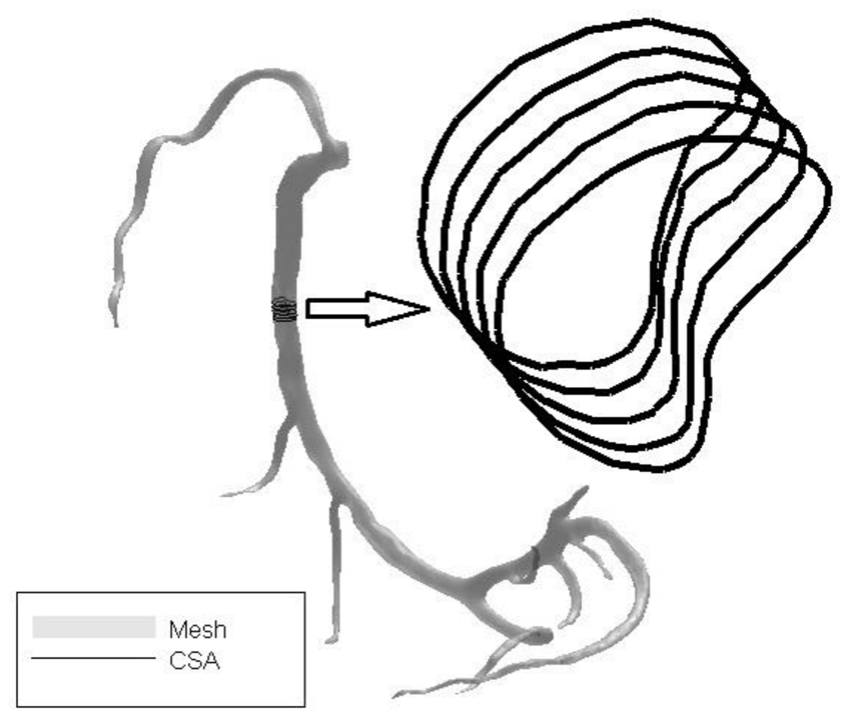
A segment of crescent shape lumen is extracted from a vessel. The CSAs are magnified as solid lines on the right side.

**Figure 4:**
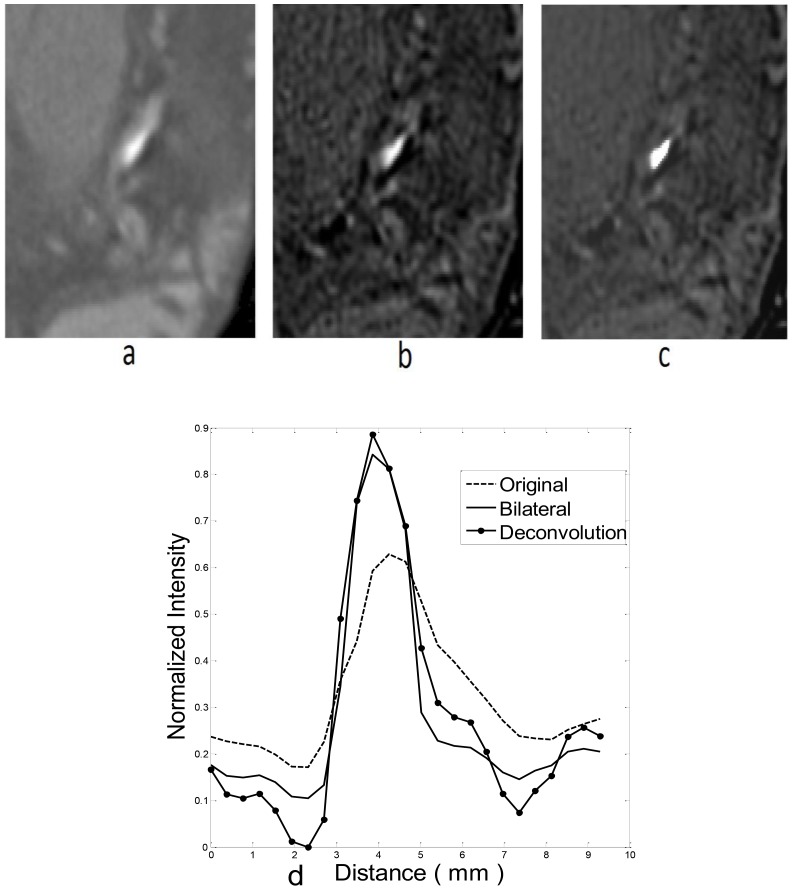
a) 2D slice from original DICOM images. **b**) Deconvolution result. **c**) Bilateral filtering result. **d**) A profile is drawn across the stenosis region and the normal vessel. Image intensity level is normalized for comparison.

An example of lumen CSA is depicted in **Figure 3** where a segment of lumen with crescent shape is shown. As the computation of CSA is independent of the actual shape of vessel, it is suitable for both circular and non-circular shape lumen. To obtain more reliable lumen geometry, the removal of CT blooming artifact is necessary. In **Figure 4b**, the deconvolution result is shown by a 2D slice image and **Figure 4c** is the result of bilateral smoothing which provides more accurate edge feature for image segmentation.

**Figure 5:**
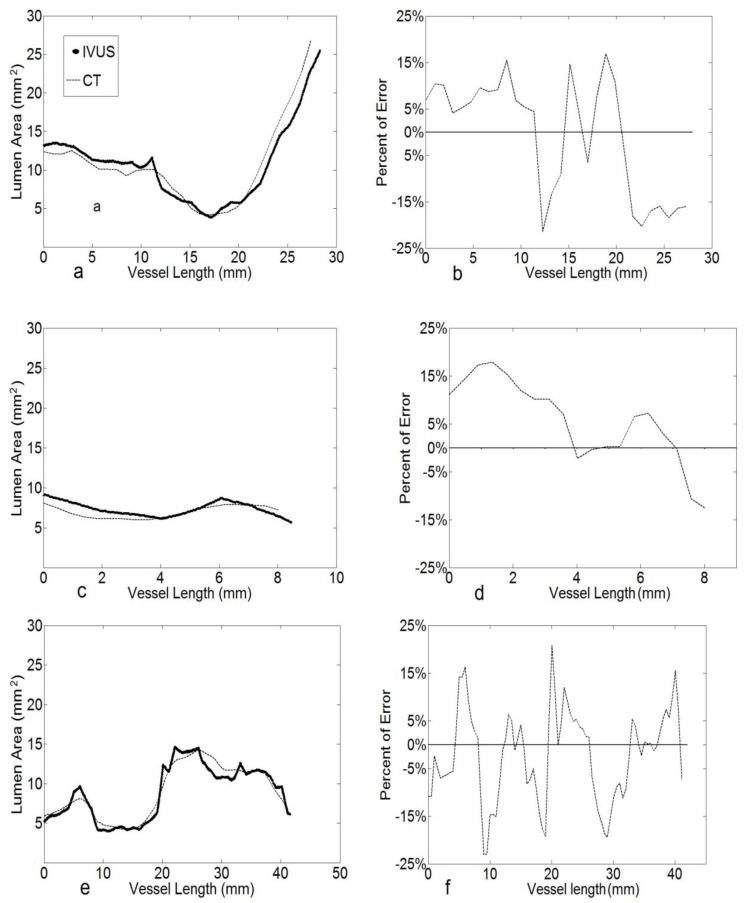
a) Area Comparison of CT and IVUS for a representative LAD. The thick dotted line the IVUS CSA and the thin dotted line is the CT data. **b**) The percent of error ((CSA_IVUS_−CSA_CT_/CSA_IVUS_)*100) for each point pairs between CT and IVUS for LAD. **c**) Area Comparison of CT and IVUS for representative LCX. **d**) The percent of error for each point pairs between CT and IVUS for LCX. **e**) Area Comparison of CT and IVUS for RCA with a stenosis at the bifurcation. **f**) The percent of error for each point pairs between CT and IVUS for representative RCA.

In **Figure 5a**, a comparison between CT and IVUS lumen area from a representative LAD is shown. The examples of IVUS data are shown in **Table 1**. **Figure 5b** shows the percent error for each point which is computed as (A_IVUS_−A_CT_)/A_IVUS_*100. The average percent error from all LAD data was 11.2%. Correspondingly, the root-mean-square error (RMSE) normalized to mean value was 13.9%. As the CSA in the stenosis segment may have a non-circular shape, the diameter was used as simple measure and computed from an inscribed circle fitted in the CSA. For the diameter, the percent of average error was 7.9%, with a RMSE of 2.5%. In **Figure 5c**, the LCX CSA is compared where the average percent error was 8.3% and normalized RMSE value was 11.2%. In **Figure 5e**, the RCA CSA has a 7.9% average error.

**Table 1:** An example of IVUS scanned that only includes eight out of more than 1600 frames from one LAD.

Frame	Position	MaxL_D_	MinL_D_	AvgL_D_	L_Area_
1	0.0167	4.26	3.98	4.13	13.2
2	0.0335	4.24	3.98	4.12	13.2
3	0.0503	4.24	3.98	4.12	13.2
4	0.0671	4.24	3.98	4.12	13.2
5	0.0838	4.24	3.98	4.12	13.2
6	0.100	4.23	3.98	4.11	13.1
7	0.117	4.23	3.98	4.11	13.1
8	0.134	4.23	3.98	4.11	13.1

*MaxL_D_, Min L_D_, AvgL_D_ and L_Area_ represent maximal, minimal, and average lumen diameter and lumen area, respectively*.

**Figure 6:**
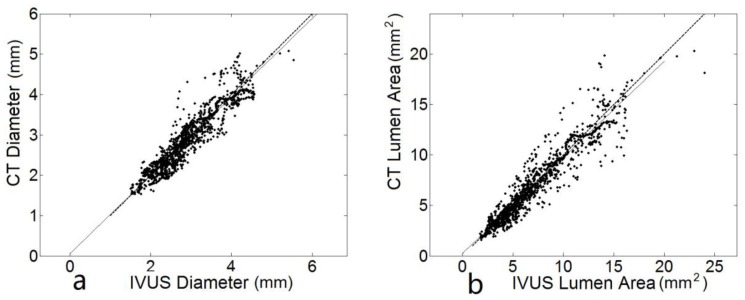
a) A linear least square fit of all data from CT and IVUS diameter: the solid dot is the scattered data pairs, the solid line is the fitted line and dotted line is the y = x function. **b**) A linear least square fit of all data from CT and IVUS CSA.

The validation data from all 11 vessels are summarized in **Figures 6a and 6b**. **Figure 6a** that shows the identity plot of diameter comparison between IVUS data CT data. The least square fit is given by y = 0.97x+0.057, the RMSE normalized to mean is 9.5%. For CSA comparison in **Figure 6b**, y = 0.95x+0.23, and the RMSE normalized to mean is 16.2%.

## Discussion

We developed a semi-automatic CT-based segmentation algorithm that provides accurate center line reconstruction for CSA data of coronary arteries including stenotic lesions (**Figures 5** and **6**) as compared with IVUS in patients (**Figure 1** and **Table 1**). The implications and limitations of the methodology are discussed below.

The angiogram has long been considered the “gold standard” for imaging of coronary arteries due to its excellent resolution. Reconstruction of 3-D images requires at least 2 orthogonal projection images [19], [20]. Jung et al. [1] used cross-sections to generate realistic geometry as coronary arteries have varying cross-sections along the vessel axis. One limitation of angiogram, however, is that it only allows visualization of the vessel lumen with no information on vessel wall and plaque structure. Ambrose et al. have shown that lipid rich vulnerable plaques are typically not significantly stenotic and often cannot be detected by angiogram [21], [22], [20]. For that reason, IVUS has become increasingly popular [23]. The use of angiogram and IVUS has often been combined to produce ANGUS (ANGiogram-IVUS) imaging [24], [25], which provides both an overall view of the vascular lumen and detailed wall structure. This combination along with blood flow measurements can further provide assessment of endothelial shear stress, an important factor in the atherosclerosis process.

Although diameter is one of the morphometric measurements typically considered, the assumption of a circular shape of the vessel is not accurate in diseased arteries. Instead, CSA is a better suited measure which is not affected by assumed shape or the severity of stenosis. The detection of lumen stenosis with non-circular geometry is one of the advantages of the present approach. As depicted in **Figure** 3, the center line is first extracted by morphological thinning which ensures the center line to be inside the vessel and independent of the non-circular shape of lumen area. As the plane is based on the center line, this intersection plane is certain to intersect with the vessel and the accuracy is not affected by the actual vessel shape. For the series of planes, the cross-section where the center point deviates can be adjusted by changing the plane's normal direction. Planes with deviated orientation can be detected by comparing the distance to other planes and adjusted if needed by resampling between neighboring planes.

Hence, we focused on direct validation of CSA in this study and found the error to be <20% (<10% error in diameter; **Figures 5** and **6**) as compared to the significantly larger error reported in Voros et al. [16]. In Voros et al's study, CT image segmentation and lumen area extraction were based on curved multi-planar reformation (cMPR) methods. In this investigation, 3D information of all branches is obtained, the vessels were segmented directly in original image slices and converted into surface mesh. The filtering on both image and geometry domain can improve the smoothness.

Given the potential artifacts resulting from the optical diffusion function, deconvolution methods are developed to separate the degraded boundary features from stenosis and normal vessels. Wiener Filters proved to be an efficient deblurring approach [26]. While high-frequency features of stenosis are improved by deconvolution, the low-density feature of surrounding tissue will be affected by noise and ring artifacts. To compensate, histogram-based selective deblurring is applied to restore high quality image by combining both original and deconvoluted images [27], [28]. But intensity histograms are based on the selection of a local region and the threshold from the local gray scale is still depended on various constitution of the surrounding tissue. In some cases, peaks and valleys in the histogram from surrounding tissues do not always provide obvious clues as to setting a threshold. In CT reconstruction, the projection of scanned data is coupled with regularization constraints to resolve the reconstruction inverse problem [29]. The purpose of the regularization can be regarded as anisotropic smoothing. We implemented similar bilateral filtering for anisotropic smoothing to restore and preserve the plaque edge [30]. Region growing and edge detection methods, like local maximal gradient [15] can be used to localize the boundary directly. As depicted in **Figure 4**, bilateral filtering results show a clear edge at the location of the stenosis.

Center line extraction also called skeleton extraction is a critical procedure for geometry reconstruction. An accurate extraction remains a challenging research topic, where a comprehensive survey can be found in Cornea et al. [31]. The methods of diameter or CSA computation can be separated into four categories: derivative-based, threshold-based, densitometry, and model-based techniques [32]. For stenosis, the category should be considered based on accuracy.

The geometric extraction methods are divided into image domain or geometry domain. Direct description of an object in image domain implies the image element is on volume grid, and the accuracy is on the level of a discrete pixel value. The disadvantage is that there is position deviation of half a voxel, at most. If an object is represented in the geometry domain, or triangle mesh domain, the accuracy is improved by converting the data into a continuously determined value. The center line jaggedness problem can only be eliminated in the geometry domain. Jaggedness is the typical problem in skeleton thinning algorithms [33]–[39]. Jaggedness is demonstrated visually in **Figure S1a**. The error is derived from numeric representation, which is clearly illustrated at the large curvature positions. To overcome the problem, a smoothness processing was applied to thinned results and then interpolation was implemented on the thinned points [14].

The Bézier curve served as both interpolation and a filtering function. Bézier curve control points do not always lie on the curve which can make the jagged center line smoother. Over-smoothness caused by the Bézier curve is modified by a local Bezier curve. Besides the image volume domain computation, the use of a mesh surface to obtain the center line is another preferable method if there is a directional computation that can be pointed to center points. The well-known mesh contraction method uses the normal vector [40]. A similar method can be found in Nordsletten et al. [41] and Wischgoll et al. [15] which uses a vector or vector field analysis to localize the center point. The CSA can be obtained in the same process, but there are gaps among some center points. In this study, morphometric refinement computations were done in simpler and direct processing steps. The initial center line can be a discrete value, and it is easy to convert it into a continuous value.

Some limitations of the current approach are noteworthy. First, the proposed CSA extraction can only process a straight vessel without bifurcation and it is necessary to remove branches from the target artery by manual operation or by computer automation. For those vessel segments near a bifurcation, the removal of one a branch can be made based on the segmentation results. The stenosis in the interested vessel of interest can be retained, and the center line can still be refined using a Bézier curve to obtain CSA. Although bifurcations are eliminated in determination of CSA, this step does not affect the accuracy of the entire vessel. Second, the starting points in IVUS do not always coincide accurately with the CT image and this can result in length misalignment. The same problem can be found at some end points, where the ratio of difference between CT and IVUS is larger than those from other segments. The angiograms were used to better match the start and end points of IVUS corresponding to CT images. Finally, the majority of the computational cost stems from the segmentation steps. For high quality images, it is not difficult to distinguish the artery from other tissue. Low quality images may cause unsatisfactory segmentation results, which may be due to contrast agent density variation, insufficient image resolution, or patient physiological variability. The overlapped regions in the image require a more complicated analysis algorithm. In this study, manual intervention was used in these specific positions. A Machine Learning approach can be used for development of an automated method [12], [13], [42], but it requires a large amount of training samples for statistical analysis of local features. Some suggested approaches use classical pattern recognition technology to design segmentation tools, but the establishment of training samples is not well rooted to allow generalization of algorithms. Further improvements should rely on the optimized feature selection and should be undertaken in the future studies.

## Conclusions

This study validated morphometric data from CT images based on IVUS. The proposed method of CSA extraction is accurate for 3D geometric reconstruction including stenosis in coronary arteries. The CT geometric reconstructions can be used to construct mathematical models for biomechanical simulation.

## Supporting Information

Appendix S1 (DOCX)

Figure S1 **a) Circle dotted line is the result of thinning algorithm without filtering. Solid circle dotted line is the thinned with filtering result.** The thin solid line is the Bézier result from unfiltered thinned line. The thick solid line represents the Bézier result from filtered thinned line. Square region is the surface mesh. **b**) The improvement of center line extraction from initial estimation. Bézier curve is shown as a solid line, and the center line is the dotted line. CSA is the solid polygon with an asterisk at its center point. **c**) Comparison between partly interpolation and full interpolation. **d**) 3D geometry of RCA, three CSAs are overlaid on surface mesh, middle one is near the bifurcation. **e**) The reconstructed CSA in segment of vessel from **d**, three CSAs are indicated in thick lines and positions are marked by circles in the curve of lumen area of that segment. This RCA vessel corresponds to curve in Figure 5e.
(DOCX)

## References

[1] Jung J, Lyczkowski RW, Panchal CB, Hassanein A (2006) Multiphase hemodynamic simulation of pulsatile flow in a coronary artery. Journal of biomechanics 39: 2064–73. 16111686 10.1016/j.jbiomech.2005.06.023

[2] Salzar RS, Thubrikar MJ, Eppink RT (1995) Pressure-induced mechanical stress in the carotid artery bifurcation: a possible correlation to atherosclerosis. Journal of biomechanics 28: 1333–40. 8522546 10.1016/0021-9290(95)00005-3

[3] Simmons CA, Grant GR, Manduchi E, Davies PF (2005) Spatial heterogeneity of endothelial phenotypes correlates with side-specific vulnerability to calcification in normal porcine aortic valves. Circ Res 96: 792–9. 15761200 10.1161/01.RES.0000161998.92009.64 PMC3057118

[4] Stein PD, Hamid MS, Shivkumar K, Davis TP, Khaia F, Henry JW (1994) Effects of cyclic flexion of coronary arteries on progression of atherosclerosis. The American journal of cardiology 73: 431–7. 8141082 10.1016/0002-9149(94)90671-8

[5] Thubrikar MJ, Baker JW, Nolan SP (1988) Inhibition of atherosclerosis associated with reduction of arterial intramural stress in rabbits. Arteriosclerosis, Thrombosis, and Vascular Biology 8: 410–20. 10.1161/01.atv.8.4.410 3395277

[6] Cebral JR, Lohner R, Soto O, Yim PJ (2001) On the modeling of carotid artery blood flow from magnetic resonance images. ASME-PUBLICATIONS-BED 50: 619–20.

[7] Taylor CA, Draney MT, Ku JP, Parker D, Steele BN, et al (1999) Predictive medicine: computational techniques in therapeutic decision-making. Computer Aided Surgery 4: 231–47. 10581521 10.1002/(SICI)1097-0150(1999)4:5<231::AID-IGS1>3.0.CO;2-Z

[8] Wang KC, Dutton RW, Taylor CA (1999) Improving geometric model construction for blood flow modeling. Engineering in Medicine and Biology Magazine, IEEE 18: 33–9. 10.1109/51.805142 10576070

[9] Calvo B, Pena E, Martinez M, Doblaré M (2007) An uncoupled directional damage model for fibred biological soft tissues. Formulation and computational aspects. International journal for numerical methods in engineering 69: 2036–57.

[10] Holzapfel GA, Gasser TC, Ogden RW (2000) A new constitutive framework for arterial wall mechanics and a comparative study of material models. Journal of elasticity 61: 1–48.

[11] LaDisa JF, Olson LE, Molthen RC, Hettrick DA, Pratt PF, et al (2005) Alterations in wall shear stress predict sites of neointimal hyperplasia after stent implantation in rabbit iliac arteries. American Journal of Physiology-Heart and Circulatory Physiology 288: H2465–H75. 15653759 10.1152/ajpheart.01107.2004

[12] Florin C, Paragios N, Williams J (2005) Particle filters, a quasi-monte carlo solution for segmentation of coronaries. Medical Image Computing and Computer-Assisted Intervention–MICCAI 2005: 246–53. 10.1007/11566465_31 16685852

[13] Lacoste C, Finet G, Magnin IE Coronary tree extraction from X-ray angiograms using marked point processes. Proc. Biomedical Imaging: Nano to Macro, 2006. 3rd IEEE International Symposium on, 2006:157–60: IEEE

[14] Zhang L, Chapman BE, Parker DL, Roberts JA, Guo J, et al (2005) Automatic detection of three-dimensional vascular tree centerlines and bifurcations in high-resolution magnetic resonance angiography. Investigative radiology 40: 661–71. 16189435 10.1097/01.rli.0000178433.32526.e0

[15] Wischgoll T, Choy JS, Ritman EL, Kassab GS (2008) Validation of image-based method for extraction of coronary morphometry. Annals of biomedical engineering 36: 356–68. 18228141 10.1007/s10439-008-9443-x

[16] Voros S, Rinehart S, Qian Z, Vazquez G, Anderson H, et al (2011) Prospective validation of standardized, 3-dimensional, quantitative coronary computed tomographic plaque measurements using radiofrequency backscatter intravascular ultrasound as reference standard in intermediate coronary arterial lesions: results from the ATLANTA (Assessment of Tissue Characteristics, Lesion Morphology, and Hemodynamics by Angiography With Fractional Flow Reserve, Intravascular Ultrasound and Virtual Histology, and Noninvasive Computed Tomography in Atherosclerotic Plaques) I study. JACC: Cardiovascular Interventions 4: 198–208. 21349459 10.1016/j.jcin.2010.10.008

[17] Koo B-K, Yang H-M, Doh J-H, Choe H, Lee S-Y, et al (2011) Optimal intravascular ultrasound criteria and their accuracy for defining the functional significance of intermediate coronary stenoses of different locations. JACC: Cardiovascular Interventions 4: 803–11. 21777890 10.1016/j.jcin.2011.03.013

[18] Lorensen WE, Cline HE Marching cubes: A high resolution 3D surface construction algorithm. Proc. ACM Siggraph Computer Graphics, 1987, 21:163–9: ACM

[19] Ladak HM, Milner JS, Steinman DA (2000) Rapid 3D segmentation of the carotid bifurcation from serial MR images. ASME J Biomech Eng 122: 96–9. 10.1115/1.429646 10790835

[20] Moore J, Steinman D, Prakash S, Johnston K, Ethier C (1999) A numerical study of blood flow patterns in anatomically realistic and simplified end-to-side anastomoses. Journal of biomechanical engineering 121: 265. 10396691 10.1115/1.2798319

[21] Alderman EL, Corley SD, Fisher LD, Chaitman BR, Faxon DP, et al (1993) Five-year angiographic follow-up of factors associated with progression of coronary artery disease in the Coronary Artery Surgery Study (CASS). Journal of the American College of Cardiology 22: 1141–54. 8409054 10.1016/0735-1097(93)90429-5

[22] Ambrose JA, Tannenbaum MA, Alexopoulos D, Hjemdahl-Monsen CE, Leavy J, et al (1988) Angiographic progression of coronary artery disease and the development of myocardial infarction. Journal of the American College of Cardiology 12: 56–62. 3379219 10.1016/0735-1097(88)90356-7

[23] Nissen SE, Yock P (2001) Intravascular ultrasound: novel pathophysiological insights and current clinical applications. Circulation 103: 604–16. 11157729 10.1161/01.cir.103.4.604

[24] Krams R, Wentzel J, Oomen J, Vinke R, Schuurbiers J, et al (1997) Evaluation of endothelial shear stress and 3D geometry as factors determining the development of atherosclerosis and remodeling in human coronary arteries in vivo Combining 3D reconstruction from angiography and IVUS (ANGUS) with computational fluid dynamics. Arteriosclerosis, thrombosis, and vascular biology 17: 2061–5. 10.1161/01.atv.17.10.2061 9351372

[25] Laban M, Oomen J, Slager C, Wentzel J, Krams R, et al. ANGUS: A new approach to three-dimensional reconstruction of coronary vessels by combined use of angiography and intravascular ultrasound. Proc. Computers in Cardiology 1995, 1995:325–8: IEEE

[26] Gonzalez RC, Woods RE, Eddins SL (2009) Digital image processing using MATLAB. Gatesmark Publishing Knoxville

[27] Rollano-Hijarrubia E, Manniesing R, Niessen WJ (2009) Selective deblurring for improved calcification visualization and quantification in carotid CT angiography: Validation using micro-CT. Medical Imaging, IEEE Transactions on 28: 446–53. 10.1109/TMI.2008.2006529 19244016

[28] Rollano-Hijarrubia E, van der Meer F, van der Lugt A, Weinans H, Vrooman H, et al. Improving the imaging of calcifications in CT by histogram-based selective deblurring. Proc. Medical Imaging, 2005:67–78: International Society for Optics and Photonics

[29] Do S, Karl WC, Liang Z, Kalra M, Brady TJ, Pien HH (2011) A decomposition-based CT reconstruction formulation for reducing blooming artifacts. Phys Med Biol 56: 7109. 22025109 10.1088/0031-9155/56/22/008

[30] Tomasi C, Manduchi R Bilateral filtering for gray and color images. Proc. Computer Vision, 1998. Sixth International Conference on, 1998:839–46: IEEE

[31] Cornea ND, Silver D, Min P (2007) Curve-skeleton properties, applications, and algorithms. Visualization and Computer Graphics, IEEE Transactions on 13: 530–48. 10.1109/TVCG.2007.1002 17356219

[32] Hoffmann KR, Nazareth DP, Miskolczi L, Gopal A, Wang Z, et al (2002) Vessel size measurements in angiograms: a comparison of techniques. Medical physics 29: 1622. 12148745 10.1118/1.1488603

[33] Bertrand G, Aktouf Z A three-dimensional thinning algorithm using subfields. Proc. Proceedings of the SPIE conference on Vision Geometry, 1994:113–24:

[34] Brunner D, Brunnett G Mesh segmentation using the object skeleton graph. Proc. Computer Graphics and Imaging, 2004, 2004:48–55: ACTA Press

[35] Gong W, Bertrand G A simple parallel 3D thinning algorithm. Proc. Pattern Recognition, 1990. Proceedings., 10th International Conference on, 1990, 1:188–90: IEEE

[36] Lohou C, Bertrand G (2004) A 3D 12-subiteration thinning algorithm based on *P*-simple points. Discrete Applied Mathematics 139: 171–95.

[37] Palágyi K, Kuba A Directional 3D thinning using 8 subiterations. Proc. Discrete Geometry for Computer Imagery, 1999:325–36: Springer

[38] Palágyi K, Kuba A (1999) A parallel 3D 12-subiteration thinning algorithm. Graphical Models and Image Processing 61: 199–221.

[39] Tsao YFF (1981) K.S (1981) A parallel thinning algorithm for 3D pictures. Comput Vis Graph Image Process 17: 315–31.

[40] Au OKC, Tai CL, Chu HK, Cohen-Or D, Lee TY Skeleton extraction by mesh contraction. Proc. ACM Transactions on Graphics (TOG), 2008, 27:44: ACM

[41] Smith N (2006) Structural morphology of renal vasculature. Am J Physiol Heart Circ Physiol 291. 10.1152/ajpheart.00814.2005 16399870

[42] Zheng Y, Loziczonek M, Georgescu B, Zhou SK, Vega-Higuera F, Comaniciu D Machine learning based vesselness measurement for coronary artery segmentation in cardiac CT volumes. Proc. SPIE Medical Imaging, 2011:79621K-K-12: International Society for Optics and Photonics

