# Supplementary material for: IVUS Validation of Patient Coronary Artery Lumen Area Obtained from CT Images
Source: PLoS One. 2014 Jan 29;9(1):e86949. doi: 10.1371/journal.pone.0086949 (PMC3906085; doi:10.1371/journal.pone.0086949)
Supplement: Appendix S1 — (DOCX) [file pone.0086949.s001.docx]

**Appendices**

**CT IMAGE SEGMENTATION**

Image feature definition of coronary arteries is fundamental for image processing. It is difficult to set a simple global grayscale threshold for the region growing algorithm. The contrast or grayscale feature variation inside the vessel is a major issue in CT images. Since the CT imaging relies on injection of intravenous dye (iodine) as a contrast agent, image quality is reduced with decreased density of the contrast agent, as seen in stenosis. Additionally, there is overlap with respect to intensity values of vessels with surrounding tissues or the background. Without additional intervention, the stenosis segmentation cannot be easily distinguished from other tissue.

In this study, CT image segmentation is based on local features. The initial automatic processing is complemented by manual modification. Automated methods tend to not fully define and recognize the optimal local features. By localization at several slices, some seed points are inputted manually or by computer processing. As high intensity levels inside the vessels are stable near the skeleton region, initial seeds can be located there so that even with a higher threshold, the skeleton region can be extracted. Region growing was then used to refine the object region by checking the local histogram. The local histograms were computed from the pixels within a sphere, where the sphere was centered on these initial seeds. By dividing the histogram bins into target object and non-target object, feature-centers were formed and used in a further feature-clustering algorithm.

From an image processing point of view, a stenosis region falls outside the normal lumen intensity of CT. Higher intensity values may result from the formation of calcium deposits, and lower intensity may stem from a fatty substance in the vessel [[1](#_ENREF_1)]. In this study, the stenosis was extracted by using similar segmentation techniques compared to the vessel segmentation.

**CENTER LINE EXTRACTION**

After the segmentation steps, binary representation was used for further geometric analysis. Standard visualization methods, such as Marching Cubes [[2](#_ENREF_2)] or triangulation, converted the vessel surface into a triangle mesh. CSA computation was the basis for all morphometric values, such as vessel diameter, which can be approximated as a maximal inscribed circle in the CSA, whereas the CSA center represents a point on the skeleton.

The center line was obtained by a thinning algorithm. This classical approach saved computational time as only the discrete value was required [[3](#_ENREF_3)]. The typical center line consists of 3D points along the vessel’s axis, represented in discrete form and located on the image volume grid. Even though the phenomenon of jaggedness is a potential limitation of this discrete form, accuracy was improved by using a smoothness filter and 3D interpolation. Although average value filtering was applied on the interpolation of the center points, the curve points were still on the discrete voxel grid. A Bézier curve interpolation was then applied to produce more continuous curve points (a sample vessel is shown in **Figure A1**). Thus, the interpolated center line represents the center line more accurately at a sub-voxel level. The accuracy of the normal vector was further improved because the normal vector was also based on new sub-voxel points rather than jagged discrete points.

**PROGRESSIVE CROSS SECTIONAL AREA COMPUTATION**

The CSA extraction is not a one-step procedure as the interpolation method can only give an initial estimation of the center line. In **Figure A1a**, the thinning results without filtering are in circle marks, and the solid circle curve represents the filtered results. Both are processed by interpolation separately and the result is demonstrated in thick lines in **Figure A1a**. The vessel segment was selected from the entire object and the surface was represented as mesh triangles. The jagged discrete form was easily found near the large curvature position. Since computing a Bézier curve is the same as filtering, the Bézier curve was computed directly based on the discrete thinned points.

To approximate the real center line, further improvement was based on the initial CSA and the initial 3D geometric center was shown in **Figure A1b**. From the figure, it is clear that the initial center line is not close to the object’s axial skeleton. The solid circle is the initial CSA computed from the initial center line. The geometric center is denoted as an asterisk symbol, and it is located outside the solid line. This center point can be treated as a candidate of a new center line point. When two new neighbor center points are obtained, the direction of these two points can be approximated as a normal vector of a new CSA. These steps were repeated until the center line point converged. The progressive procedure produced the final center line, denoted by the dashed dark line.

A series of CSAs are shown in **Figure A1e** where the stenosis is near a bifurcation. A portion of the CSA was overlaid in **Figure A1e** to demonstrate the stenosis. The even distribution of distance and orientation degree in the CSA results can be used to verify CSA quality. The algorithm for computing the CSA requires the computation of the intersection plane between the normal vector plane of one center point and all triangle mesh surface, which is converted into computing intersection lines between the plane and one 3D triangle. The theoretical foundation can be found in Dan Sunday‘s website [[4](#_ENREF_4)].

**References**

1. Kelm B, Mittal S, Zheng Y, Tsymbal A, Bernhardt D, et al (2011) Detection, grading and classification of coronary stenoses in computed tomography angiography. *Medical Image Computing and Computer-Assisted Intervention–MICCAI 2011*:25-32

2. Lorensen WE, Cline HE Marching cubes: A high resolution 3D surface construction algorithm. *Proc. ACM Siggraph Computer Graphics*, *1987*, 21:163-9: ACM

3. Lee TC, Kashyap RL, Chu CN (1994) Building skeleton models via 3-D medial surface/axis thinning algorithms. *CVGIP: Graphical Model and Image Processing* 56:462-78

4. Sunday D *Geometry Algorithms Home*. <http://geomalgorithms.com/index.html>
