# Supplementary figures and images for: IVUS Validation of Patient Coronary Artery Lumen Area Obtained from CT Images
Source: PLoS One. 2014 Jan 29;9(1):e86949. doi: 10.1371/journal.pone.0086949 (PMC3906085; doi:10.1371/journal.pone.0086949)

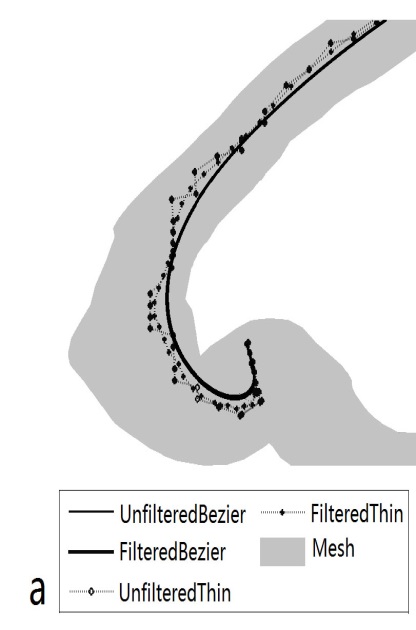

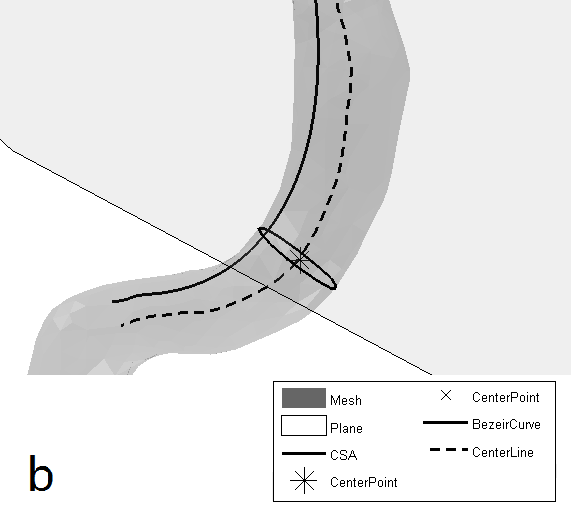

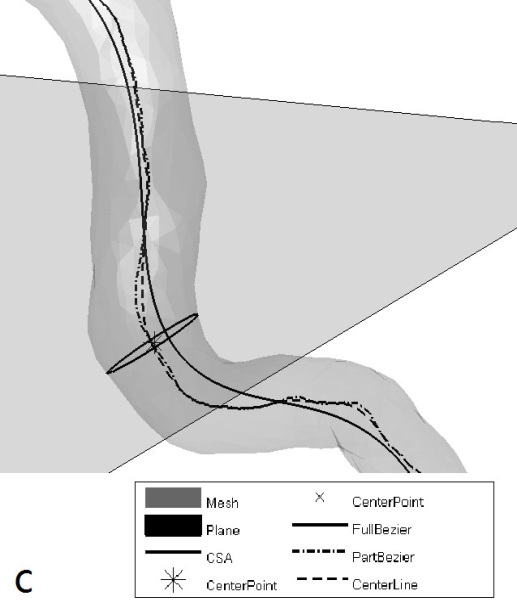

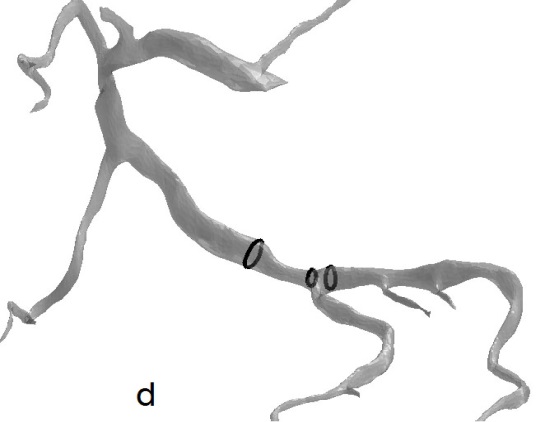

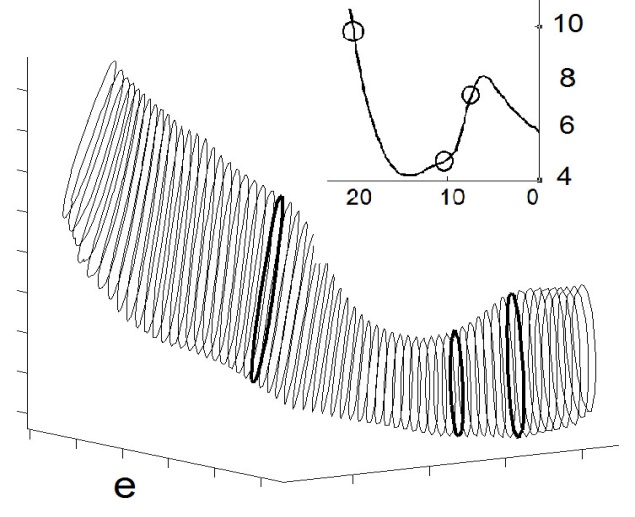


**Figure A1**

Supplement: Figure S1 — a) Circle dotted line is the result of thinning algorithm without filtering. Solid circle dotted line is the thinned with filtering result. The thin solid line is the Bézier result from unfiltered thinned line. The thick solid line represents the Bézier result from filtered thinned line. Square region is the surface mesh. b) The improvement of center line extraction from initial estimation. Bézier curve is shown as a solid line, and the center line is the dotted line. CSA is the solid polygon with an asterisk at its center point. c) Comparison between partly interpolation and full interpolation. d) 3D geometry of RCA, three CSAs are overlaid on surface mesh, middle one is near the bifurcation. e) The reconstructed CSA in segment of vessel from d, three CSAs are indicated in thick lines and positions are marked by circles in the curve of lumen area of that segment. This RCA vessel corresponds to curve in Figure 5e. (DOCX) [file pone.0086949.s002.docx]
